# Effect of Miracle Berry on Taste Modification Properties Among Adults Living in Australia: A Multi‐Phase Study Protocol

**DOI:** 10.1002/fsn3.71640

**Published:** 2026-03-12

**Authors:** Getahun Fentaw Mulaw, Shashya Diyapaththugama, Chris Irwin, Natalie Shilton, Indu Singh, Rati Jani

**Affiliations:** ^1^ School of Pharmacy and Medical Sciences Griffith University Gold Coast Queensland Australia; ^2^ Department of Public Health College of Health Sciences, Woldia University Woldia Ethiopia; ^3^ School of Health Sciences and Social Work Griffith University Gold Coast Queensland Australia

**Keywords:** food preferences, miracle berry, natural low‐caloric sweetener, taste perception

## Abstract

Miracle Berry (MB) contains miraculin, a glycoprotein that alters sour tastes into sweet. MB may enhance the taste perceptions of hypo‐palatable foods (e.g., sour/bitter fruits and vegetables) and hyper‐palatable foods (e.g., sugar‐sweetened beverages (SSBs)), potentially influencing their liking and intake. Therefore, this study aims to assess the effects of different doses of MB on the taste perception, overall liking and intake of hypo‐palatable foods and hyper‐palatable SSBs. Additionally, it will explore the feasibility of MB in enhancing food preferences, energy intake, and dietary quality in a natural environment. The study consists of four phases. Phase 1 will employ a three‐arm pre‐post design to assess the effect of different doses of MB on the taste perception of individual food‐based solutions and single food items (measured using the generalized labeled magnitude scale, gLMS) in 54 healthy‐weight adults. Phases 2 and 3 will utilize a four‐arm, placebo‐controlled, pre‐post trial to determine the effective dose of MB that could change the taste perception (using gLMS), overall liking (using the labeled affective magnitude scale) and intake of hypo‐palatable mixed foods and hyper‐palatable SSBs in 40 healthy‐weight and 40 overweight/obese adults, respectively. Phase 4 will be a 12‐week, two‐arm, placebo‐controlled feasibility trial assessing the effect of MB on food preferences (food liking questionnaire), energy intake (3‐day food record and 24‐h dietary recall), dietary quality (Australian Recommended Food Score) and other feasibility indicators in 30 overweight/obese adults. Outcomes of phase 4 will be assessed at baseline, Week 1, Week 6, and Week 12. The effects of MB will be evaluated using mixed‐effects models, and a post hoc power analysis will be conducted.

## Graphical abstract

Miracle Berry ( *Synsepalum dulcificum* ) (MB), through its active glycoprotein miraculin, transforms sour tastes into sweet ones. This four‐phase quasi‐experimental protocol involves healthy‐weight (Phases 1 and 2) and overweight/obese (Phases 3 and 4) adults to examine the taste modification properties of MB. The phases progress from testing MB's effects on individual food‐based solutions and items to mixed foods and beverages, and finally to evaluating its feasibility in improving food preferences, energy intake, and dietary quality in real‐life settings.

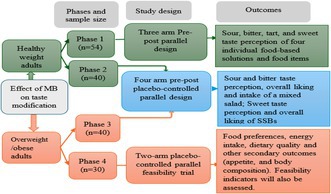

**Abbreviations**
ABS: Australian Bureau of Statistics
ARFS: Australian Recommended Food Score
BIA: Bioelectrical Impedance Analysis
BMI: Body Mass Index
EFSA: European Food Safety Authority
FDA: Food and Drug Administration
FFM: Fat‐free Mass
FLQ: Food Liking Questionnaire
gLMS: Generalized Labeled Magnitude Scale
HPLC: High Performance Liquid Chromatography
IPAQ: International Physical Activity Questionnaire
LAMS: Labeled Affective Magnitude Scale
MB: Miracle Berry
NCDs: Non‐communicable Diseases
PROP: 6‐n‐propylthiouracil
PTC: Phenylthiocarbamide
SSBs: Sugar Sweetened Beverages
TAS2R38: Taste Receptor type 2 member 38
WC: Waist Circumference
WHtR: Waist‐to‐Height Ratio

## 1 Introduction

Overweight and obesity are significant global public health issues (Global Burdon of Disease Collaborators 2017), and are major contributors to the rise of non‐communicable diseases (NCDs) such as Type 2 diabetes and other cardiovascular diseases (Goossens 2017; Tutor et al. 2023). The global economic burden of overweight and obesity is projected to exceed US$3 trillion annually by 2030 (World Health Organization (WHO) 2024).

Diet is a major modifiable determinant of overweight and obesity (Di Ciaula and Portincasa 2019) and NCDs (Al‐Jawaldeh and Abbass 2022); in particular, excessive intake of sugar‐rich foods and poor intake of core foods such as fruit and vegetables. Eating habits are shaped by a combination of biological (i.e., genetic) and environmental determinants, with taste preference being one of the primary motivating factors (Boesveldt et al. 2018). Humans have an innate liking for sweet‐tasting foods and a general aversion to sour and bitter tastes (Beauchamp 2016). This is often reflected in national food and nutrient consumption data, with large proportions of dietary energy intake coming from foods considered to be of little nutritional value (e.g., sugar‐sweetened beverages, SSBs). The data also suggest that the majority of the population consumes inadequate amounts of fruit and vegetables (Australian Bureau of Statistics 2022).

Adopting strategies that influence eating behaviors, perceptions, and food preferences is essential to effectively change dietary habits and improve dietary intake (i.e., reducing sugar consumption and increasing fruit and vegetable intake) (Mattei and Alfonso 2020; Pinho et al. 2018). While nutrition education has been widely used to address this issue, it often requires individualized counseling and has proven largely inefficient and unsustainable (Katenga‐Kaunda et al. 2022; Vasiloglou et al. 2019).

Non‐nutritive (low‐calorie) artificial sweeteners may be useful as a means of reducing sugar (and its associated energy) intake while maintaining flavor profiles (Sievenpiper et al. 2025). Many non‐nutritive sweeteners (NNS) are available (e.g., saccharin, aspartame, cyclamate, and acesulfame‐K) as low‐calorie alternatives to sugar (Mora and Dando 2021). Although NNS are approved as food additives and possess strong sweetness‐inducing potential (Samreen and Dhaneshwar 2023), their use may be accompanied by undesirable effects such as unpleasant aftertaste and intolerance symptoms (e.g., headache, nausea, vomiting, diarrhea, mood changes) (Ali et al. 2021; Bernardo et al. 2016). While NNS are considered safe within acceptable daily intake limits, uncertainties remain regarding their long‐term health consequences (Lohner et al. 2017). Meta‐analysis indicates that although few randomized controlled trials (RCTs) demonstrated modest short‐term benefits of NNS for weight reduction, findings from long‐term cohort studies suggest possible associations with adverse outcomes, including increased body mass index (BMI), Type 2 diabetes, and cardiovascular disease (Rios‐Leyvraz et al. 2022). Consequently, the WHO (2023) recommends limiting NNS use for weight management in favor of dietary strategies that naturally enhance sweetness. Regulatory bodies such as the U.S. Food and Drug Administration (FDA 2025) and the European Food Safety Authority (EFSA 2025), continue to affirm the safety of NNS within established limits, while acknowledging uncertainties regarding their long‐term effects. Additionally, artificial sweeteners primarily enhance sweetness and preserve palatability (Sievenpiper et al. 2025), but they are ineffective in reducing aversive taste qualities, such as sourness and bitterness. This limitation underscores the need for natural alternatives that not only enhance sweet taste perception but also mitigate these aversive attributes. Miraculin, in particular, exhibits this dual functionality and requires further investigation.

Miraculin is an active glycosylated protein extracted from the pulp of miracle berry (MB), also known as the miracle fruit ( *Synsepalum dulcificum* Daniell), a member of the Sapotaceae family (Misaka 2013). It comprises two glycosides attached to a single polypeptide chain with 191 amino acid residues (Kabore et al. 2022). Due to this active protein, MB has the unique ability to temporarily alter taste perception, making sour foods taste sweet (Diyapaththugama et al. 2024). This effect occurs from miraculin binding to taste receptors on the tongue and modifying how they respond to sour stimuli (Sanematsu et al. 2016). As a result, foods that are typically acidic (i.e., sour) can taste sweet after ingesting MB (Misaka 2013). Our previous review demonstrated that MB consistently decreases sourness and enhances sweetness in sour‐tasting solutions and food items. However, findings regarding its effects on bitter, salty, and sweet tastes remain inconsistent (Diyapaththugama et al. 2024). These discrepancies may stem from variations in experimental conditions, particularly considering that miraculin requires a pH range of 4.8–6.5 for activation (Akinmoladun et al. 2020; Doddawad et al. 2022).

The taste modification property of MB has generated considerable interest due to its potential to influence overall liking of hypo‐palatable fruits and vegetables, hyper‐palatable SSBs, and mixed meals. By reducing the aversive qualities of sour and bitter tastes, MB may increase the preference for such fruits and vegetables. Indeed, evidence indicates that MB enhances the overall liking of individual sour‐tasting foods (Choi and Garza 2021; Choi and Park 2023); however, its effect on the preference and intake of other hypo‐palatable foods with a bitter taste profile is yet to be investigated. Beyond sour and bitter foods, MB has been shown to enhance the sweetness of already sweetened binary and trinary mixtures (Capitanio et al. 2011), which may result in a sensation of oversweetness. Consumer research suggests that when sweetness surpasses optimal levels and reaches the rejection threshold, preference and intake decline, potentially contributing to lower sugar consumption (Peng et al. 2023). Despite this, no study has examined the role of MB on changes to the overall liking of hyperpalatable SSBs. Furthermore, since most foods are consumed as mixed meals, it is notable that only one randomized cross‐over trial has investigated the effect of MB on the overall liking of mixed meals (Choi and Park 2023). In this study, a 400 mg dose of MB was selected based on its previously demonstrated effectiveness in individual sour‐tasting foods (Choi and Garza 2020, 2021). The same dose significantly increased liking for breakfast and dinner meals, which were specific to Korean‐American cultural diets, but showed no significant effect for lunch. The authors suggested that the effective dose of MB for modifying taste perception and overall liking may vary depending on the type of food. To the best of our knowledge, no dose‐comparison study has been conducted on taste perception, overall liking and intake for mixed meals.

MB, as a potential NNS, also shows promise as a natural sugar substitute, with implications for reducing energy intake (Rodrigues et al. 2016). In a randomized, placebo‐controlled crossover trial among prediabetic and diabetic individuals, MB consumption lowered total energy intake by approximately 441 kJ compared with placebo (Choi and Park 2023). However, these findings were derived under controlled experimental conditions in which participants were provided with standardized meals, thereby limiting generalisability to real‐life settings. In addition, as MB has been reported to reduce the aversive perception of sour and bitter tastes (Andrade et al. 2019; Rodrigues et al. 2016), it may help facilitate healthier dietary patterns by improving the palatability of nutrient‐dense but under‐consumed sour and bitter fruits and vegetables. Collectively, these potential roles, attenuating energy intake and improving fruit and vegetable consumption, suggest that MB has potential to make a meaningful contribution to overall diet quality. However, to date, no study has examined these effects in real‐life settings.

Overall, as this project focuses on the taste‐modifying properties of MB, it is important to consider factors that may affect taste perception. Individuals with overweight or obesity often exhibit altered taste perception compared with those of healthy weight (Hajimaghsoodi et al. 2025; Peinado et al. 2023). These differences may influence the role of MB in modifying taste perception and food liking. To address this, the project is structured into four phases: the first two involve healthy‐weight adults to establish baseline effects of different MB doses on taste perception and liking, while the latter phases focus on adults with overweight or obesity, a population often encouraged to reduce added sugar intake and increase fruit and vegetable consumption (Koliaki et al. 2018). Furthermore, because both fruits and vegetables (Arias et al. 2022) and MB (Mosquera et al. 2025) possess antioxidant properties, assessing feasibility in adults with overweight or obesity may also provide additional health benefits, given their elevated risk of oxidative stress (Jakubiak et al. 2021).

## 2 Aims and Objectives

This project aimed to investigate the taste modification properties of MB in adults living in Australia, addressing the following general objectives:

- Assess the effect of different doses of MB on sour, bitter, tart, and sweet taste perception of individual food‐based solutions and food items in healthy‐weight adults living in Australia (Phase 1).
- Compare the effect of different doses of MB on sourness and bitterness taste perceptions, the overall liking and intake of hypo‐palatable mixed food, and the sweetness taste perception and overall liking of hyper‐palatable SSBs in healthy‐weight (Phase 2) and overweight/obese (Phase 3) adults living in Australia.
- Investigate the feasibility of using MB as a potential natural low‐calorie sweetener to modify food preferences, energy intake, and overall dietary quality in overweight/obese adults living in Australia (Phase 4).

## 3 Methods and Materials

### 3.1 Project Phases and Study Design

Each phase of the project will use different quasi‐experimental designs. Table 1 presents a summary of the study design, population, setting, variables, sample size, and study period for each phase.

**TABLE 1:** Study designs, study populations, study settings, study variables, sample size and estimated study periods for each phase of the project.

	Project phases			
	Phase 1	Phase 2	Phase 3	Phase 4
Study design	Three‐arm pre‐post parallel design	Four‐arm placebo‐controlled single‐blind pre‐post parallel design	Four‐arm placebo‐controlled single‐blind pre‐post parallel design	Two‐arm placebo‐controlled, single‐blind (participants), parallel feasibility trial
Study population	Healthy‐weight adults living in Australia	Healthy‐weight adults living in Australia	Overweight or Obese adults living in Australia	Overweight or Obese adults living in Australia
Study setting	Laboratory‐based	Laboratory‐based	Laboratory‐based	In a natural environment (at home)
Independent variable	Different MB doses (175, 350, and 700 mg)	Different MB doses (175, 350, and 700 mg) and placebo	Different MB doses (175, 350, and 700 mg) and placebo	MB or Placebo consumption in a natural environment
Dependent variable	Sour, bitter, tart, and sweet taste perception ratings of food‐based solutions and individual food items	Ratings for sourness and bitterness taste perceptions, overall liking and intake of hypo‐palatable mixed vegetarian salad Ratings for sweetness taste perceptions and overall liking of SSBs	As mentioned in Phase 2	Primary outcomes Food preferences (Overall meal liking and preferences across time) Energy intake Dietary quality Other outcomes of interest Feasibility of the treatment protocol and study procedures Appetite Anthropometry/Body composition
Sample size	54	40	40	30
Proposed study period	1 July–30 September, 2024	1 November 2024–30 September 2025	1 December–30 June, 2025	1 May–30 December, 2025

*Note:* Sociodemographic characteristics (sex*, age, income, employment status, and education status), phenotypic taste perception, genotypic taste sensitivity, mouth pH, anthropometry, and body composition will be collected as covariates. In Phases 3 and 4, comorbidities (e.g., diabetes, hypertension) and medication use (self‐reported) will also be assessed. In Phase 4, physical activity will be measured as a covariate for secondary outcomes. *Sex is classified based on biological characteristics.

### 3.2 Nutritional Profile of MB and Placebo Tablets

For this project, Sen Yuh Farm Science Co. Ltd., Taiwan (https://www.mberrytw.org) donated the MB and placebo tablets. MB tablets consisted of dried MB pulp (55%) and corn starch (45%) as a binding agent. Each tablet weighs 350 mg and contains approximately 0.16 mg of miraculin, according to the donor's report. This content of miraculin is within the range limit of 0.052%–0.32% used previously (Greenaway et al. 2024). The placebo tablets are composed of dragon fruit (20%) and corn starch (80%). Given concerns about the potential toxicity of synthetic food colorants and the corresponding recommendation to favor natural alternatives (Amchova et al. 2024; Malabadi et al. 2022), dragon fruit was incorporated into the placebo formulation to naturally match the color of the MB tablets and thereby support blinding. Table S1 presents the nutritional profile of the MB tablets provided by the donor organization, and the sugar analyses (glucose, fructose, sucrose, maltose, and mannose) of both MB and placebo tablets. Sugar analyses were conducted at the Queensland Alliance for Agriculture and Food Innovation (QAAFI), Centre for Nutrition and Food Sciences (https://qaafi.uq.edu.au/), using an in‐house method with high‐performance liquid chromatography (HPLC). As indicated in Table S1, the analysis indicated that the placebo tablets contained low levels of glucose and fructose, with no detectable sucrose, maltose, or mannose. This finding aligns with existing evidence showing that dragon fruit contains only minimal amounts of glucose and fructose and no detectable sucrose or maltose (Kakade et al. 2022; Tarte et al. 2023), while corn starch contains negligible sugar (Zhang et al. 2021). Therefore, the contribution of the 20% dragon fruit to the overall sugar content of the placebo tablets is considered minimal.

### 3.3 Eligibility Criteria

The inclusion and exclusion criteria for all project phases are summarized in Table 2. Rubino et al. (2025) reported that Body Mass Index (BMI) alone is insufficient to define obesity, as it does not differentiate fat mass from lean mass. The recommendations are to confirm obesity through direct measurement of body fat where possible; otherwise, it should be defined as a high BMI combined with at least one additional adiposity indicator (waist circumference [WC], waist‐to‐height ratio [WHtR], or waist‐to‐hip ratio), or the presence of two such indicators irrespective of BMI. In line with this recommendation, we plan to use the WHO age‐, sex‐, and ethnicity‐specific cut‐offs (Barba et al. 2004; Li et al. 2023; Potter et al. 2024; WHO 2008). For this study, a healthy weight is defined as a BMI of 18.5 – 24.9 kg/m^2^ for Europeans and others, or 18.5 – 22.9 kg/m^2^ for Asian populations. Overweight/obesity is defined as a BMI ≥ 25 kg/m^2^ for Europeans and others, or ≥ 23 kg/m^2^ for Asian populations, together with at least one additional criterion: elevated WC (≥ 94 cm for men, ≥ 80 cm for women, ≥ 90 cm for Asian men), WHtR ≥ 0.5, or body fat percentage ≥ 25% for men or ≥ 35% for women, or meet at least two of the additional adiposity criteria. Detailed sex‐ and ethnicity‐specific cut‐offs to be used in Phases 3 and 4 are provided in Table S2.

**TABLE 2:** Inclusion and exclusion criteria in each phase of the project.

Criteria	Phases 1 and 2	Phases 3 and 4
Inclusion criteria	Adults living in Australia Age 18–65 years Healthy weight BMI	Adults living in Australia Age 18–65 years BMI in the Overweight/Obesity category plus ○ High WC or high WHtR OR ○ High body fat percentage
Exclusion criteria	Known alterations to the sense of smell and taste, including dysphagia SARS‐CoV‐2 infection within the last 3 months Food allergies or intolerances to berries. Current pregnancy or lactation (Turck et al. 2021) Self‐reported non‐communicable disease (Cancer, diabetes, hypertension, and chronic/acute liver disease or kidney problems)	All exclusions proposed for Phases 1 and 2 will apply, except those related to medical conditions. Participants with self‐reported cancer or chronic or acute liver or kidney problems will be excluded. However, those with other stable medical conditions (e.g., controlled diabetes, controlled hypertension, and other chronic diseases) will be eligible to participate in Phases 3 and 4. For Phase 4, as it is a follow‐up study requiring participants to take at least two MB/placebo tablets daily for 3 months, participants with existing medical conditions will be advised to consult their physician prior to confirming enrolment, in case they have unstable conditions (e.g., uncontrolled diabetes, uncontrolled hypertension, or other critical illnesses)

*Note:* Exclusion criteria are based on potential alterations to taste perception that may bias outcome assessment (Bernhardson et al. 2008; Boltong and Keast 2012; Brennan et al. 2020; Duffy 2020; Feng et al. 2014; Kanjanaumporn et al. 2020; Risso et al. 2020).

### 3.4 Sample Size

Table 1 summarizes the sample size for each phase and is determined based on previous effect estimates (Phase 1), the number of fixed‐effect factors to be considered in the regression model (Phases 2 and 3), and the recommended minimum sample size per treatment arm for feasibility studies (Phase 4, Table 2).

#### 3.4.1 Phase 1

The sample size is calculated, considering an effect estimate (Cohen's d) of 0.78 from Wong and Kern (2011) on the sweetness taste perception obtained before and after taking MB using G*power software version 3.2, considering 80% power, α level of error of 0.05, and 20% dropout. Using paired sample *t*‐tests, 54 participants (18 in each group) will be recruited.

#### 3.4.2 Phases 2 and 3

Determining sample size for mixed models (LMM/GLMM) is challenging, as statistical power depends on multiple factors, including the number of participants, repeated measures per subject, intra‐class correlation (ICC), outcome distribution, and the specified random‐effects structure (Kumle et al. 2021). Given uncertainties, particularly regarding predictors that will ultimately be retained as fixed effects in the final model, the sample size for this study is determined using an empirically informed approach. Consistent with Green (1991) guidelines for regression analyses, for behavioral research with expected large effect estimates, a minimum of 10 participants per predictor variable will be required for included to ensure model stability. In the planned mixed model, treatment (i.e., dose) will be specified as the primary fixed effect, time (pre‐post assessment) as the within‐subject factor, and participant as a random effect. Covariates such as age, sex, and phenylthiocarbamide (PTC) sensitivity will be considered for inclusion to control for potential confounding but will only be retained in the final model if they contribute meaningfully to model stability and fit. Based on an anticipated inclusion of four fixed factors in the final model, a minimum of 40 participants will be recruited for each study phase.

#### 3.4.3 Phase 4

Whitehead et al. (2016) recommend feasibility trial sample sizes of 75, 25, 10, and 10 per arm for standardized effect sizes categorized as extra small (< 0.1), small (0.1–0.29), medium (0.3–0.69), and large (≥ 0.7) in a main trial with 80% power and 5% significance. In a randomized crossover trial, Choi and Park (2023) reported medium‐to‐large effect sizes for MB in improving the overall liking of both breakfast (pumpkin oatmeal with banana and blueberries) and dinner (a Korean‐style Baekban meal with rice, soup, and side dishes) in prediabetic and diabetic adults. Therefore, considering a medium to large effect estimate, a minimum of 20 participants (10 per arm) would be sufficient for this feasibility trial. However, in a previous exploratory three‐arm trial evaluating the efficacy and safety of miraculin in 31 cancer patients, attrition of 32% was observed over a three‐month follow‐up (López‐Plaza et al. 2024). To account for this, up to 30 participants (15 per arm) will be recruited. This will satisfy the median sample size target for feasibility studies (Totton et al. 2023).

### 3.5 Participant Recruitment

Participants will be recruited using a convenience sampling technique. Recruitment will be advertised through Griffith University volunteer research broadcasts, social media platforms, and printed flyers displayed in various locations, including Griffith University premises (Gold Coast campus), medical centres, community centres, restaurants, places of worship, and leisure centres. Flyers will include the principal investigator's contact details (email) and a QR code for registration. In addition, recruitment strategies will include radio advertisements, direct in‐person/word‐of‐mouth approaches, and invitations to students enrolled in courses at Griffith University, Gold Coast campus. The recruitment plan is presented in Table S3.

### 3.6 Data Collection Tools

Table 3 describes measurement tools for each key outcome variable and covariates in each project phase.

**TABLE 3:** Measurement tools and their description for key variables in each phase of the project.

Phases	Variable to be measured		Tool	Description	References
Phase 1	Sour, bitter, tart and sweet taste perceptions of individual food‐based solutions and food items		Generalized Labeled Magnitude Scale (gLMS)	Ranges from 0 (barely detectable) to 100 (the strongest sensation of any kind). Participants will establish these boundaries (no sensation to strongest imaginable) before rating items	Green et al. (1996)
Phases 2 and 3	Overall liking of hypo‐palatable mixed vegetarian salad and hyper‐palatable SSB		Labeled Affective Magnitude Scale (LAMS)	Ranges from 0 (greatest imaginable dislike) to +100 (greatest imaginable like). Study participants will be asked, “How much do you like/dislike this food item? (please put a slash (/) mark somewhere on the line).”	Schutz and Cardello (2001)
	Sourness and bitterness taste perceptions of hypo‐palatable mixed vegetarian salad and sweetness taste perceptions of hyper‐palatable SSBs		Generalized Labeled Magnitude Scale (gLMS)	Ranges from 0 (barely detectable) to 100 (the strongest sensation of any kind). Before rating items, participants will establish these boundaries (no sensation to the strongest imaginable)	Green et al. (1996)
	Intake of the hypo‐palatable mixed vegetarian salad		The difference between the amount served and the amount left over	At both the pre‐test and post‐test sessions, participants will be provided with a fixed portion of mixed salad weighing 117 g, which corresponded approximately to half a serve of vegetables and half a serve of fruit according to the Australian Dietary Guidelines. Participants will be instructed to consume as much of the provided salad as they desired. Each portion will be weighed prior to serving, and any leftovers will be weighed immediately after consumption. Actual intake will be calculated as the difference between the amount served and the amount remaining	National Health and Medical Research Council (2013)
Phase 4	Change in the food preferences in a natural environment (i.e., at home)		Food‐liking questionnaire (FLQ)	FLQ is validated for the Australian population and contains 73 food items. It measured liking using a nine‐point hedonic scale, in which ‘like extremely’ was coded as ‘9’ and ‘dislike extremely’ was coded as ‘1’. If participants have never eaten a particular food or never experienced one of the listed items, they will be instructed to rate the item as “neither like nor dislike”. At baseline, sixth‐ and 12th‐week participants will score their liking of all listed food items in the FLQ. In the FLQ foods, items are grouped into 10 main categories based on the Australian Guide to Healthy Eating: grains, vegetables, fruits, dairy, animal‐based protein, plant‐based protein, fat and oil, sweet food, salty food, and alcohol. The mean in food‐liking scores for each food category will be generated (Wanich et al. 2020). Finally, the change in mean liking score for each food group, and the total change in food liking score, will be analyzed across measurement sessions	Wanich et al. (2018)
	Change in the overall liking of the specific or mixed food as a meal (breakfast, lunch, dinner, snack) in a natural environment (i.e., at home)		Any perceived change will be recorded using a yes/no response, and its magnitude will be quantified using Labeled Affective Magnitude Scale (LAMS)	Participants will record whether there is a perceived change in overall liking of meals due to the tables (yes/no). Then using LAMS, in which 0 represents the greatest imaginable dislike, and + 100 represents the greatest imaginable like, they will rate their liking of that specific meal. The scale will include options to select the meal type (breakfast, lunch, dinner, snack), a space to note down the major meal constituents, a space to attach photos of the meal (photo of a meal before and after eating, and the date and the time the meal was consumed)	Schutz and Cardello (2001)
	Perceived change in appetite		Perceived change in appetite (yes, no), and the direction of change (increase appetite or decreased appetite)	Along with the change in overall liking of meals, participants will note the effect of the tablet on their appetite (whether it helps them eat less, causes no change, or makes them eat more)	
	Change in energy intake		A 3‐day food record method	Dietary intake will be collected at baseline, sixth, and 12th weeks. Then, the quantity and type of food items consumed will be changed to calorie intake and macronutrient intake (carbohydrates, proteins, total fat, saturated fat) using the AUSNUT 2011–2013 food composition database (Grech et al. 2017)	Bailey (2021)
			24‐h dietary recall method	At each visit, the 3‐day food record and the Australian Recommended Food Score (ARFS) will be supplemented with the 24‐h dietary recall (24 h) method to comprehensively assess dietary intake. The 24HR will be conducted through structured interviews to collect detailed information on all foods and beverages consumed in the preceding 24 h. Portion sizes will be estimated using food models, household measures, or standardized references, and nutrient intake will be analyzed using the AUSNUT 2011–2013 food composition database. Dietary quality indices will be determined by categorizing consumed foods into predefined food groups. To account for variability in dietary intake, two non‐consecutive 24HRs, one on a weekday and one on a weekend day, will be collected at baseline, week 6, and week 12	Castell et al. (2015)
	Change in dietary quality		Australian Recommended Food Score (ARFS) questionnaire	The ARFS is derived from the Australian Eating Survey food frequency questionnaire but focuses on the 70 questions related to nutrient‐dense core foods as classified by the Australian Dietary Guidelines. The ARFS includes eight sub‐scales: vegetables (*n* = 21), fruit (*n* = 12), meat (*n* = 7), non‐meat/flesh protein foods (*n* = 6), breads and cereals (*n* = 13), dairy foods (*n* = 11), water (*n* = 1), and spreads and sauces (*n* = 2). The total ARFS score ranges from 0 to 73, calculated by summing the points for each food item from different food groups. Changes in the total ARFS score or changes in the scores of the eight food groups will be compared between the MB and placebo groups	Collins et al. (2014, 2015)
	Physical activity as a covariate		International Physical Activity Questionnaire (IPAQ)	Physical activity will be measured using the short form of the International Physical Activity Questionnaire (IPAQ). The short, self‐administered format is designed to assess physical activity during the previous 7 days across four domains: vigorous activity, moderate activity, walking, and sitting time. It consists of seven questions and will provide estimates of the frequency (days per week) and duration (minutes per day) of activities	Craig et al. (2003)
All phases	Anthropometry	Height	Portable stadiometer (Model No. Seca 213)	The stadiometer will be mounted upright on a labeled, hard surface. Participants will take off their shoes and socks and stand straight, with their heads aligned in the Frankfort plane and their heels, buttocks, upper back, and occiput touching the stadiometer	Kobel et al. (2022); WHO (1995)
		Waist circumference	Non‐stretchable measuring tape (seca‐201 Tape)	Measured at the midpoint between the iliac crest and lower costal border, just above the level of the umbilicus. The subject stands with arms folded across the chest and is measured at the end of normal expiration. Tapes will be regularly inspected to ensure it is not bent or damaged.	Mason and Katzmarzyk (2009)
	Body composition (Body weight, BMI, Fat mass, fat‐free mass, body fat percentage, body water)		Bioelectrical impedance analysis (BIA) (Model No. BC‐541)	BIA measures body composition by sending a small electric current (50 kHz) through the body and measuring the tissue resistance encountered (Marra et al. 2019). The assumption is that the current travels more easily through lean tissue containing more water than fat tissue. It is valid in estimating body fat percentage (R^2^ = 0.92 compared to DXA) (von Hurst et al. 2016). In this study, we will use BIA Model No. BC‐541, the person will stand barefoot on the device's footplates and grasp the handles. Before attending the lab, participants will be instructed to refrain from consuming alcohol and avoid vigorous physical exercise (24 h) and not eat or drink anything 1 h before the test. Within the lab, participants will be asked to remove all metal objects and jewelry, and they will be asked if they have any electrical devices, like a pacemaker. For measuring weight, participants will be instructed to wear light clothing and remove footwear	Kyle et al. (2004)
	Phenotypic taste sensitivity		PTC paper strips	Measured by placing PTC strips on the tongue. Individuals who report the PTC strip as bitter will be categorized as tasters, and those who do not taste bitterness will be categorized as non‐tasters (Veluswami et al. 2015)	Veluswami et al. (2015)
	Genotypic taste sensitivity		*TAS2R38* gene from saliva	Saliva samples will be collected using OG‐500 Oragene•Dx kits (2 mL). Participants will rinse their mouths with water before sampling (Garbieri et al. 2017). TAS2R38 gene will be measured via PCR after DNA extraction. Common haplotypes such as PAV (Proline‐Alanine‐Valine), AVI (Alanine‐Valine‐Isoleucine), and PAV/AVI will be identified (Negri et al. 2015)	Robino et al. (2022)
	pH of the mouth		pH paper strip	Participants will spit their saliva into a disposable cup provided and be guided to place the pH paper strip in the saliva. Then, the researcher will determine the pH based on the color change of the pH paper	Aoyama et al. (2017)
	Sociodemographic variables		Sociodemographic questionnaire	Age, sex, income, educational status, and occupational status will be collected using a sociodemographic questionnaire based on the Australian Bureau of Statistics. The questionnaire will incorporate associated comorbidities and the type of current medication intake	ABS (https://www.abs.gov.au/)

### 3.7 Data Collection Procedure

#### 3.7.1 Standardization

For standardization, the following instructions will be sent to participants 24‐h prior to their scheduled visit (Phases 1–3). These procedures are intended to minimize variability in factors that may influence taste perception, food liking, appetite, and subsequent food intake.

- Do not feel overly full or very hungry before attending the lab (Fu et al. 2021).
- Refrain from eating or drinking (except water) 1 h before attending the lab (McCrickerd et al. 2015).
- Do not brush your teeth 1 h before attending the lab (Choi and Garza 2020).
- Do not chew gum 1 h before attending the lab (Miquel‐Kergoat et al. 2015).
- Refrain from drinking alcohol for at least 8 h before attending the lab (Marinho Brazil et al. 2022).
- Do not smoke 1 h before attending the lab (Kale et al. 2019).
- Avoid strenuous physical activities 24 h before attending the lab (Douglas et al. 2017; Martins et al. 2015).
- Do not participate if you have experienced unexpected, sad/shocking, or happy events in the past 24 h. In such situations, participants will be allowed to book another day when their mood status returns to normal (Evers et al. 2018).

#### 3.7.2 Phase 1

Food‐grade solutions representing each taste will be prepared, as outlined in Table 4. Upon arrival at the laboratory, participants will be asked to review the standardization instructions provided before their visit and confirm their adherence by ticking the relevant checklist.

**TABLE 4:** Food‐grade solutions and food items with their concentrations and pH values representing each taste (Phase 1).

Taste type	Food‐grade solutions		Food items	
	Amount, concentration	pH	Amount	pH
Sour	10 mL solution of 10 mmol/L citric acid	2.60	Piece of lime (1/8th lime or ~12 g)	2.50
Bitter	10 mL of 3.2 mmol/L PROP	6.80	Broccoli floret (~20 g)	6.92
	5 mL of 10 mmol/L citric acid +5 mL of 3.2 mmol/L of PROP	2.70	5 mL of lime juice + Broccoli floret (~20 g)	4.00
Tart	10 mL of 7.5 mmol/L tartaric acid	3.78	Piece of Granny Smith apple without seeds (1/8th apple or ~18 g)	3.14
Sweet	10 mL of 96 mmol/L sucrose	7.00	5 mL maple syrup	6.78
	5 mL of 10 mmol/L citric acid +5 mL of 96 mmol/L sucrose	3.02	5 + 5 mL of lime juice + maple syrup	3.27

*Note:* For non‐acidic solutions and food items (bitter and tart), we planned to use the solutions mixed with citric acid, as an acidic pH was essential for the taste modification action of miraculin (Doddawad et al. 2022). Food samples were kept in a refrigerator, then removed and placed on separate plastic trays 10 min before each test.

**FIGURE 1:**
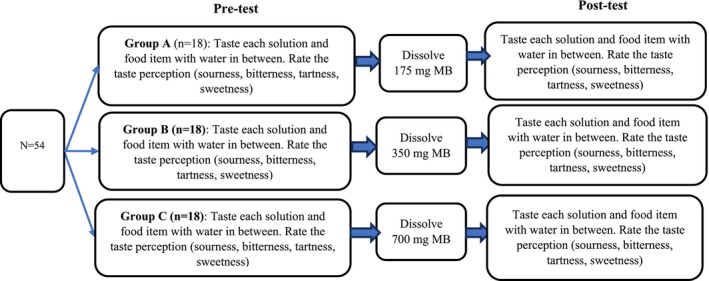
Phase 1 study design.

First, sociodemographic characteristics and anthropometric measurements will be collected. Saliva samples will then be obtained for TAS2R38 genotyping, and oral pH and phenotypic taste sensitivity will be assessed. As illustrated in Figure 1, participants (*N* = 54) will subsequently be allocated into three groups (*n* = 18 per group). Prior to commencing the pre‐test session, participants will be instructed to drink a sip of water and wait at least 30 s (Choi and Garza 2020). In their respective groups, participants will receive 10 mL of each solution and pieces of single food items to rate their perceived taste perception on a generalized labeled magnitude scale (gLMS). Participants will rinse their mouths with water between each solution or food item until the previous taste stimulus is gone (Igarashi et al. 2013; Lipatova and Campolattaro 2016).

After the pre‐test, participants will drink water to neutralize their mouths. Group A will receive 175 mg (half a tablet), Group B will receive 350 mg (one tablet), and Group C will receive 700 mg (two tablets) doses in MB tablets. Subjects will be instructed to dissolve the tablet with saliva on their tongue for 5 min and make contact with the tablet against as much surface area of the oral cavity as possible without chewing or swallowing it (Hudson et al. 2018). Immediately after dissolving, participants will receive the same solutions and food items used for the pre‐test to capture changes in perceived taste perception on gLMS.

#### 3.7.3 Phase 2

A hypo‐palatable vegetarian salad containing half‐serve vegetables (raw broccoli and rocket leaves) and half‐serve fruits (Granny Smith apple), which are the minimum possible servings per Australian dietary guidelines (Brownie et al. 2015), will be prepared 30 min before the data collection time at Griffith University, Gold Coast campus kitchen lab or the Participants' home, based on the participants' preferences. Food items for the vegetarian salad and the Coca‐Cola (representing hyper‐palatable SSB) will be purchased from the local market. The food will be stored in a refrigerator (4°C), removed, and placed on separate plastic trays 10 min before each test. The SSB will be kept at room temperature, and 30 mL will be poured into 50 mL paper cups, which are clean and uniform in color (Mielby et al. 2018).

**FIGURE 2:**
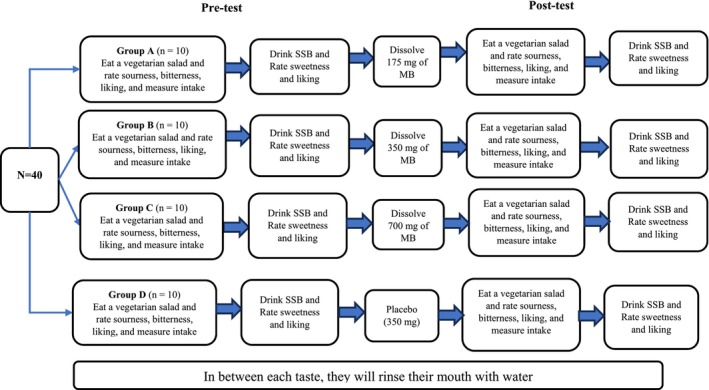
Phases 2 and three study design.

Figure 2 shows that participants (*n* = 40) will be allocated to four treatment arms (*n* = 10 in each group). The data collection procedure will follow these steps:

1. Initially, participants will review the participant information sheet and screening questions and sign a consent form. They will confirm (tick) the standardized instructions provided before their visit.
2. Anthropometry measurements and body composition assessments will be conducted, and eligibility will be confirmed.
3. For the TAS2R38 gene, saliva will be collected using the Oragene DNA OG‐500 kit as mentioned in Table 3.
4. The pH of the mouth will be measured using a pH paper strip as mentioned in Table 3.
5. Phenotypic taste sensitivity will be assessed by having participants place a PTC strip on the tip of their tongues.
6. Rate their hunger level using the 10‐point hunger scale (0 = full, and 10 = very hungry), and assess their current mood status using a 10‐point mood scale (0 = happiness, and 10 = sadness).
7. After rinsing their mouths with water, participants will receive a weighted vegetarian mixed salad. They will consume as much or as little from the given salad.
8. Participants will rate the sourness and bitterness taste perception of the salad using gLMS, followed by their overall liking of the salad using the Labeled Affective Magnitude Scale (LAMS). The principal investigator will measure and record any leftover salad to measure their intake.
9. After rinsing their mouths with water, participants will drink 30 mL of regular Coca‐Cola and rate its sweetness‐taste perception on gLMS and overall liking on LAMS.
10. Participants will be required to wait for a compulsory 20‐min period after the pre‐testing procedures before commencing post‐testing. This interval is intended to allow hunger levels to return to baseline. The duration is based on the estimated energy content of the test meal (60 kcal, i.e., 251 kJ). Drawing on Horner et al. (2014), whose participants required approximately 3 h to recover from a ~1600 kJ meal, we calculated that ~23 min would be sufficient for our test meal; therefore, a minimum 20‐min waiting period will be adopted. As this duration may not fully account for individual differences in metabolism, dietary fiber intake, or the amount consumed during the pre‐test, participants will be verbally informed of the rationale for the interval and asked to notify the investigator once their hunger has returned to baseline. They will then be asked to complete the same hunger scale used upon arrival at the lab. If post‐waiting hunger ratings are comparable to baseline, the post‐test will proceed; otherwise, the waiting period will be extended until hunger levels return to baseline.
11. According to their assigned group, participants will rinse their mouths and dissolve a given dose of MB (175, 350, 700 mg) or placebo (350 mg) in their mouth for 5 min.
12. A vegetarian salad similar in content and amount to the one provided in the pre‐test will be provided. Participants will consume it as desired, and the principal investigator will measure and record any leftovers.
13. Participants will rate the sourness and bitterness taste perception of the salad using gLMS, followed by their overall liking using LAMS.
14. Finally, participants will rinse their mouths with water, drink 30 mL of regular Coca‐Cola, and rate the sweetness taste perception using gLMS and their overall liking using LAMS.

#### 3.7.4 Phase 3

The study design and data collection procedures followed in this phase are identical to those used in Phase 2 (Figure 2). They vary among study participants (Phase 2 is on healthy‐weight adults, and Phase 3 is on overweight or obese adults). A separate investigation is required because taste sensitivity alters with overweight and obesity (Harnischfeger and Dando 2021; Rohde et al. 2020; Vignini et al. 2019). Therefore, the dose of MB required to induce a change in taste perception and food liking may be different for overweight and obese adults than for healthy‐weight adults.

#### 3.7.5 Phase 4

This will be a two‐arm, placebo‐controlled, single‐blind, parallel feasibility trial conducted over a 12‐week follow‐up period, in which participants will be blinded to group allocation. Blinding of participants will be maintained via the use of MB and Placebo tablets of similar size and closely matched colourings. Most importantly, participants will only receive their allocated treatment tablets and will not be privy to viewing the alternative treatment tablet. To minimize the risk of unblinding within households, only one participant per household will be enrolled. As this is a feasibility study, the success of blinding will also be evaluated by asking participants at the end of the trial whether they believed they had received MB or placebo.

The participants will have four visits to the Gold Coast campus, or they will be visited at their home by the principal investigator, based on their preference. That is, a first visit (at baseline), a second visit (at the end of the baseline week), a third visit (at the end of the 6th week), and a fourth visit (at the end of the 12th week). The main outcomes of interest are food preferences, energy intake, and dietary quality. Additionally, it will explore its effect on the overall liking of mixed meals (breakfast, lunch, dinner, and snacks) and on appetite. As it is a feasibility study, the other main outcomes of interest will be exploring the feasibility indicators (recruitment, blinding, allocation, retention, compliance, and adverse effects). Furthermore, as a secondary outcome, the study will investigate the effect of MB on anthropometry and body composition. Table 5 indicates the details of the study variables to be measured, including their frequency and the study visits at which they will be assessed.

**TABLE 5:** Variables to be measured at each visit and throughout the follow‐up time of the Phase 4.

Variables to be measured at each visit	Intervention time and follow‐up duration													
	First visit	Second visit						Third visit						Fourth visit
	Day zero	Baseline week (week 0)	1st week	2nd week	3rd week	4th week	5th week	6th week	7th week	8th week	9th week	10th week	11th week	12th week
Sociodemographic characteristics (age, sex, income, educational status)	●													
Phenotypic taste sensitivity (using PTC strip)	●													
Food preferences using the Food Liking Questionnaire (FLQ)	●							●						●
Dietary quality using the Australian Recommended Food Score (ARFS)	●							●						●
Energy/food intake (using a 24‐h dietary recall)	●							●						●
Energy/food intake (using a 3‐day food record)^a^		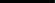						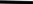						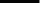
Overall liking of meals (breakfast, lunch, dinner, and snack), and appetite^b^			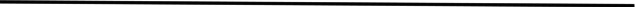											
Anthropometry	●							●						●
Body composition	●							●						●
Physical activity (IPAQ)	●							●						

^a^ The 3‐day dietary intake measurements will be supplemented with the 24‐h dietary recall method, conducted at baseline, midline, and end line. To measure dietary intake, participants will complete a 3‐day dietary intake record questionnaire within the marked week (on two weekdays and one weekend day).

^b^ Participants will record their overall liking of meals and their effect on their appetite on the Qualtrics survey link (or the hard copy version of it) throughout the 12‐week follow‐up. They will be requested to attach a photo of the meal tried with MB/placebo (both the meal served before eating and the leftover meal after eating).

##### 3.7.5.1 First Visit (Baseline Measurements)

At the first visit, baseline characteristics such as sociodemographic characteristics, phenotypic taste sensitivity, and physical activity will be measured. Food preferences will be measured using a validated Food Liking Questionnaire (FLQ), and dietary quality will be measured using the validated Australian Recommended Food Score (ARFS). Energy intake will be assessed using 3‐day food records supplemented with 24 h dietary recalls. To minimize errors in portion size estimation, participants will be instructed in the use of standardized and household measures. During the 24 h recall, participants will provide a detailed account of all foods and beverages consumed the preceding day, including brand names, ingredients, condiments, cooking methods, and portion sizes. Following the initial recall, the data collector (principal investigator) will probe for any omitted items, clarify portion sizes, and review the record jointly with the participant to ensure accuracy, with modifications made as required.

At the same visit, participants will be provided with a 3‐day food record sheet and instructed to record their intake over three non‐consecutive days (two weekdays and one weekend) during the following week. To improve accuracy, they will be encouraged to record portion sizes using package information or standardized measures and, when these are not feasible, to rely on household measures. Participants will also be advised to complete the record as soon as possible after each eating occasion to minimize underreporting and improve portion size estimations. The completed records will be reviewed by the researcher at the next visit for completeness and accuracy.

Furthermore, anthropometry and body composition will be measured as secondary outcomes of interest. During this initial visit, participants will be asked to provide their preferred way of contact (phone, email, or social media) and postal address (suburb, street, and postal code) for follow‐up purposes.

##### 3.7.5.2 Second Visit (End of Baseline Week)

In this visit, the 3‐day food record sheet provided in the first visit will be collected from study participants. Then, participants (*n* = 30) will be allocated into two arms. Then, participants will be given the required number of tablets (MB or placebo) in their respective groups for 6 weeks. Moreover, they will be provided with a 3‐day food record sheet and requested to complete it in the last week of this follow‐up period, before the third. Instructions will be provided to encourage participants to use a minimum amount of lime or lime juice while they administer tablets (to activate miraculin) with their meals in their daily lives. However, unlike the lab phases, no other strict standardization procedures will be imposed, as the aim is to evaluate the feasibility and acceptability of MB use in everyday dietary contexts.

##### 3.7.5.3 Third Visit (End of the 6th Week)

During this visit, the 3‐day food record sheet, the adverse effect recording sheet, and the daily follow‐up questionnaire provided at the second visit will also be collected. Then, all outcomes of interest measured at the baseline (FLQ, ARFS questionnaires, 24‐h dietary recall, anthropometry and body composition measurement) and physical activity as a covariate will be measured. Participants in their respective groups will receive the required MB or placebo tablets for the next 6 weeks. Additionally, to measure their energy intake, they will receive the 3‐day food record sheet and be instructed to complete it in the next sixth week (at 12 weeks of intervention).

##### 3.7.5.4 Fourth Visit (End of the 12th Week)

On this visit, all measurements taken at the third visit will be measured, and the 3‐day food record sheet provided on the third visit will be collected.

##### 3.7.5.5 Parameters Monitored Daily Throughout the Intervention

###### 3.7.5.5.1 Overall Liking of Meals (Breakfast, Lunch, Dinner, and Snacks)

Perceived changes in the overall liking of meals associated with tablet intake will be assessed daily throughout the 12‐week intervention period. For each meal consumed with the tablet, participants will indicate whether they experienced a perceived change in overall liking (yes/no) and will rate their liking using the LAMS. Data will be collected via a Griffith University‐approved Qualtrics link. Participants will also record the date and time of tablet intake, the meal type (breakfast, lunch, dinner, or snack), the main constituents of the meal, and photographs of both served and leftover portions. Recording meal constituents will enable exploration of whether the effect of MB on overall liking varies across meal occasions and specific foods. Pre‐ and post‐meal photographs will provide qualitative information to support estimation of changes in food intake. Participants who prefer not to use the Qualtrics link will be provided with a hard copy version of the questionnaire containing identical items. The principal investigator will monitor submitted responses via Qualtrics daily.

###### 3.7.5.5.2 Appetite

Perceived changes in appetite associated with tablet intake will be assessed concurrently with overall liking for the same meals consumed with the MB or placebo tablet throughout the 12‐week intervention period. For each recorded meal, participants will indicate whether they experienced a perceived change in appetite (yes/no) and will specify the direction of change, indicating whether the tablet was perceived to increase or reduce food intake. Appetite responses will be collected using the same Qualtrics link (or hard copy form) and at the same meal occasion as the overall liking assessment. Detailed daily data collection procedures for the follow‐up period (Phase 4) are presented in Table S4.

##### 3.7.5.6 Feasibility Indicators and Reminders

As the main outcome of interest, the feasibility indicators, such as recruitment, blinding, allocation, retention, compliance, and adverse effects associated with the intake of the tablets, will be explored and reported (Aschbrenner et al. 2022; Donald 2018; Teresi et al. 2022). Starting from the second visit (after allocating participants to the MB and placebo groups), specific reminders will be sent through each participant's preferred contact method. Table S5 provides details on when and how these feasibility variables will be measured, as well as the types of reminders to be delivered. At the start of treatment (second visit), participants will be provided with an adverse effect recording sheet and instructed to document any adverse effects experienced during the follow‐up period (Supporting Information: Adverse Effect Recording Sheet).

### 3.8 Data Management and Analysis Plan

Data completeness, cleaning, and editing will begin with data collection and continue through data entry. Data will be entered into Epi Data Version 4.6 and analyzed using STATA Version 8 software. The data will be checked for missing values and outliers. Descriptive quantitative analysis will be used for reporting results. Categorical variables will be presented using frequencies and percentages. Based on normality assumptions, continuous variables will be reported in mean (±SD) or median (±IQR). Normality will be assessed using both graphical methods (histogram, Q–Q plot, P–P plot) and statistical testing (Shapiro–Wilk test). Homogeneity of variances will be tested using Levene's test. Treatment effects will be determined using mixed models, which account for repeated measures within individuals as well as between‐participant differences in taste perception. A detailed analysis plan for each project phase is provided in Table 6.

**TABLE 6:** Analysis plan for each analytical objective of the project.

Phases	Analytical objectives	IV	DV	Measurement scale of the dependent variable	Analysis technique
1	To investigate the effect of different doses of MB on the taste perception of sour, bitter, tart, and sweet solutions and food items among healthy‐weight Australian adults	Different MB doses ○ Dose 1: ½ tablet (≈170 mg) ○ Dose 2: 1 tablet (≈350) ○ Dose 3: 2 tablets (≈700 mg)	Sour, bitter, tart, and sweet taste perception ratings	gLMS continuous scale (0–100)	Descriptive statistics ○ Mean (±SD)‐if assumptions for normality of residuals are met, otherwise Median (±IQR) ○ Counts and frequencies Linear (LMM) or Generalized (GLMM) mixed‐effects models will be used. The dose will be included as a fixed effect, time as a within‐subject factor, and the participant will be included as a random effect, with other covariates included as appropriate to improve goodness of fit (e.g., age, sex, taste sensitivity). Parameters that yield the best model fit according to Akaike Information Criteria will be retained. An unstructured (UN) covariance matrix will be specified initially. If convergence or estimation issues occur, a simpler covariance structure, such as variance components (VC), will be applied as an alternative. No covariance structure will be specified (unstructured). To identify the best distribution (and link for GLMMs), the data type, residual plots, Shapiro–Wilk normality test, Levene's test for homogeneity of variance and Pearson's dispersion test will be used. Type III Wald *χ* ^2^ tests will be used to generate main effects *p*‐values. Dunn‐Šidák corrected pairwise comparisons will be performed to compare taste perception ratings across treatment doses. Statistical significance will be accepted as *p* < 0.05
2	To compare the effect of different doses of MB on sourness, and bitterness taste perceptions and overall liking of a hypo‐palatable mixed salad among healthy‐weight Australian adults	Different MB doses ○ Dose 1: ½ tablet (≈170 mg) ○ Dose 2: 1 tablet (≈350) ○ Dose 3: 2 tablets (≈700 mg) Placebo	Ratings for sourness and bitterness taste perception	gLMS (0–100)	As proposed in Phase 1 Additionally, as the sample size was determined using the empirical guideline of at least 10 subjects per predictor variable (Green 1991), and given the complexity in power estimation for mixed models, post hoc sensitivity power analysis will be conducted for planned comparisons. This will determine the minimum detectable effect size based on the achieved sample size and desired statistical power. Recent literature also recommends strengthening sample size justification for such approaches through sensitivity power analyses (Lakens 2022)
			Rating for overall liking	LAMS (0–100)	
			Intake	Served amount of salad minus left over	
	To compare the effect of different doses of MB on sweetness taste perceptions and overall liking of hyper‐palatable SSBs among healthy‐weight Australian adults	Different MB doses ○ Dose 1: ½ tablet (≈170 mg) ○ Dose 2: 1 tablet (≈350) ○ Dose 3: 2 tablets (≈700 mg) Placebo	Sweetness taste perception ratings	gLMS (0–100)	
			Ratings for overall Liking	LAMS (0–100)	
3	To compare the effect of different doses of MB on sourness and bitterness taste perceptions and overall liking of hypo‐palatable mixed salad among overweight or Obese Adults residing in Australia	Different MB doses ○ Dose 1: ½ tablet (≈170 mg) ○ Dose 2: 1 tablet (≈350) ○ Dose 3: 2 tablets (≈700 mg) Placebo	Ratings for sourness and bitterness taste perceptions	gLMS (0–100)	As proposed in Phase 2
			Ratings for overall liking	LAMS (0–100)	
	To compare the effect of different doses of MB on sweetness taste perceptions and overall liking of hyperpalatable SSBs among overweight/obese adults residing in Australia	Different MB doses ○ Dose 1: ½ tablet (≈170 mg) ○ Dose 2: 1 tablet (≈350) ○ Dose 3: 2 tablets (≈700 mg) Placebo	Ratings for sweetness taste perceptions	gLMS (0–100)	
			Ratings for overall Liking	LAMS (0–100)	
4	To measure the feasibility of the treatment protocol, study procedures and outcome	Treatment ○ MB ○ Placebo Follow‐up duration	Retention rate, Compliance rate, adverse effects, recruitment and allocation success rate	Frequency and descriptive explanations	Descriptive statistics ○ Mean (±SD) for normally distributed data, otherwise Median (±IQR) ○ Counts and frequencies
	To measure the effect of MB consumption on food preferences in overweight or obese adults living in their natural environment (i.e., at home).	Treatment ○ MB ○ Placebo Measurement time (baseline, mid‐protocol, completion)	Changes in food preferences	FLQ as a continuous scale (Food liking score) on three occasions (at baseline, week 6, week 12, and at the end line)	Descriptive statistics ○ Mean (+SD) for normally distributed data, otherwise Median (+IQR) ○ Counts and frequencies The treatment effect will be assessed using LMM or GLMM, as proposed in Phase 1. Given that this is a feasibility trial, a sensitivity power analysis will be conducted to determine the minimum detectable effect size based on the available sample size and the desired statistical power. The analysis will be conducted on an intention‐to‐treat basis
	To investigate the effect of MB consumption on the energy intake of overweight or obese adults living in their natural environment (i.e., at home)	Treatment ○ MB or ○ Placebo Measurement time (baseline, mid‐protocol, completion)	Changes in energy intake	Diet intake converted to kilo calories (at baseline, week 6, week 12)	
	To determine the effect of MB consumption on the diet quality of overweight or obese adults living in their natural environment (i.e., at home)	Treatment ○ MB or ○ Placebo Measurement time (baseline, mid‐protocol, completion)	Changes in diet quality score	Total ARFS score and each food group ARFS score (at baseline, week six, week 12)	
	To determine the effect of MB consumption on overall liking of mixed meals served as breakfast, lunch, dinner, and snacks in overweight or obese adults living in their natural environment (i.e., at home)	Treatment ○ MB or ○ Placebo Measurement time (daily throughout the follow‐up period)	Comparing the change in overall liking of the meal (served as breakfast, lunch, dinner, snack)	gLMS measured daily from week 2 to week 12 (0–100) Proportion (self‐reported yes/no)	

	To determine the effect of MB consumption on the appetite for different foods in overweight or obese adults living in their natural environment (i.e., at home)	Treatment ○ MB or ○ Placebo Measurement time (daily throughout the follow‐up period)	Comparing the change in appetite	Proportion (self‐reported yes/no)	
To measure the effect of MB consumption on anthropometric and body composition measurements of overweight or obese adults living in their natural environment (i.e., at home)	Treatment ○ MB or ○ Placebo Measurement time (baseline, mid‐protocol, completion)	Changes in anthropometric and body composition measures	Weight, BMI, WC, WHtR, FFM, Body Fat Percentage, Body water (at baseline, week 6, week 12)		

Abbreviations: FFM, fat‐free mass; FM, fat mass; WC, waist circumference; WHtR, Waist‐to‐height ratio.

## 4 Comments

Food liking results from a complex interplay of sensory perceptions, with taste playing a crucial role (Liem and Russell 2019). Humans have an innate preference for sweet tastes and an aversion to sour and bitter foods (Beauchamp 2016). Therefore, a practical approach to minimize sugar intake while maintaining food palatability is to provide alternative low‐calorie sweeteners (Arshad et al. 2022; Russell et al. 2021). As a potential low‐calorie sweetener, MB may alter the liking and subsequent consumption of hypo‐palatable foods (sour and bitter‐tasting fruits and vegetables) as well as hyper‐palatable foods and SSBs, potentially impacting overall dietary quality.

Several considerations should be acknowledged. Dietary data collected through self‐reported methods may be subject to recall bias, social desirability bias, and underreporting. To minimize these issues, mitigation strategies such as participant training, use of portion size estimation aids, and researcher probing will be applied during data collection. Nevertheless, the inherent limitations of self‐reported dietary methods highlight the need for cautious interpretation of the findings.

In conclusion, this project will generate new knowledge on the effects of different doses of MB on taste perception and preferences for hypo‐palatable foods and hyper‐palatable SSBs, while also providing evidence on the low‐pH requirement for its activation in both healthy‐weight and overweight/obese adults. Furthermore, it will examine the feasibility of using MB as a taste modifier to influence food preferences, energy intake, and dietary quality in a natural environment (i.e., at home). If MB demonstrates promising effects in this feasibility trial, larger‐scale studies in real‐life settings, including diverse population groups, will be needed to guide public health strategies for reducing added sugar consumption and promoting fruit and vegetable intake, which are key targets in dietary guidelines and chronic disease prevention efforts.

## Author Contributions

**Getahun Fentaw Mulaw:** investigation (equal), methodology (equal), project administration (equal), validation (equal), visualization (equal), writing – original draft (lead), writing – review and editing (equal). **Shashya Diyapaththugama:** investigation (equal), methodology (equal), project administration (equal), validation (equal), visualization (equal), writing – original draft (equal), writing – review and editing (equal). **Chris Irwin:** ethodology (equal), validation (equal), visualization (supporting), writing – review and editing (supporting). **Natalie Shilton:** investigation (supporting), methodology (supporting), project administration (supporting), supervision (equal), validation (equal), visualization (equal), writing – review and editing (equal). **Indu Singh:** investigation (equal), methodology (supporting), project administration (equal), supervision (equal), validation (equal), visualization (equal), writing – review and editing (equal). **Rati Jani:** conceptualization (lead), investigation (equal), methodology (equal), project administration (equal), supervision (lead), validation (equal), visualization (equal), writing – original draft (equal), writing – review and editing (equal).

## Funding

The authors have nothing to report.

## Ethics Statement

The protocol has been approved by the Griffith University Human Research Ethics Committee (ref no. 2024/052). Once the study is completed, the findings will be disseminated through peer‐reviewed journals and conferences.

## Conflicts of Interest

The authors declare no conflicts of interest.

## Supporting information


**Table S1:** Nutritional profile of MB and placebo to be used in this project.
**Table S2:** Sex and ethnicity‐specific cut‐off point for the Phases 3 and 4 eligibility criteria.
**Table S3:** Participants' recruitment plan.
**Table S4:** Daily follow‐up procedures in Phase 4 for monitoring tablet consumption and assessing perceived changes in overall food liking and appetite at the meal level.
**Table S5:** Feasibility indicators, how and when they will be measured, and the possible reminders to be sent (if required).

**File S2:** Adverse‐effects monitoring and documentation procedures used during Phase 4 follow‐up.

## Data Availability

Data sharing not applicable to this article as no datasets were generated or analyzed during the current study.
