# Supplementary material for: Effect of Miracle Berry on Taste Modification Properties Among Adults Living in Australia: A Multi‐Phase Study Protocol
Source: Food Sci Nutr. 2026 Mar 12;14(3):e71640. doi: 10.1002/fsn3.71640 (PMC13093657; doi:10.1002/fsn3.71640)
Supplement: Supplementary file 1 — Table S1: Nutritional profile of MB and placebo to be used in this project. Table S2: Sex and ethnicity‐specific cut‐off point for the Phases 3 and 4 eligibility criteria. Table S3: Participants' recruitment plan. Table S4: Daily follow‐up procedures in Phase 4 for monitoring tablet consumption and assessing perceived changes in overall food liking and appetite at the meal level. Table S5: Feasibility indicators, how and when they will be measured, and the possible reminders to be sent (if required). [file FSN3-14-e71640-s002.docx]

Table S1: Nutritional profile of MB and placebo to be used in this project

| Profile | MB (350 mg) | Placebo |
| --- | --- | --- |
| Energy (kcal) | 1.2 | - |
| Total carbohydrate (mg) | 278.3 | - |
| Sugars (mg) | 120.8 | - |
| Crude protein (mg) | 10.2 | - |
| Crude fat (mg) | 8.1 | - |
| Saturated fat | ND | - |
| Trans fat | ND | - |
| Cholesterol | ND | - |
| Fiber (mg) | 13.3 | - |
| Moisture (mg) | 41 | - |
| Ash (mg) | 12.6 | - |
| Sodium Chloride (mg) | 55.3 | - |
| Miraculin (mg) | ≈0.16 | - |
| Glucose (%) | 17.91 | 8.77 |
| Fructose (%) | 18.4 | 3.3 |
| Sucrose (%) | 3.27 | ND |
| Mannose | ND | ND |
| Maltose | ND | ND |
| ND: not detected per 100 grams of sample; - Not analysed for placebo | | |

Supplementary Table 2: Sex and ethnicity-specific cut-off point for the Phase 3 and Phase 4 eligibility criteria.

| Sex | Ethnicity | BMI | WC | WHtR | Body fat percentage |
| --- | --- | --- | --- | --- | --- |
| Male | Europeans and others | >25 kg/m2 | >94 cm | >0.5 cm | >25% |
|  | Asian populations* | >23 kg/m2 | >90 cm |  |  |
| Female | Europeans and others | >25 kg/m2 | >80 cm | >0.5 cm | >35% |
|  | Asian populations* | >23 kg/m2 | >80 cm |  |  |
| *South Asian, Chinese and Japanese.  Note: for Phase 3 and 4, the inclusion criteria were defined as follows: (1) meeting the BMI threshold together with at least one additional criterion (WC or WHtR); or (2) meeting the body fat percentage criterion; or (3) fulfilling both WC and WHtR criteria irrespective of BMI. | | | | | |

Table S3: Participants’ recruitment plan

| **Sources** | **Online Flyer** | **Hardcopy flyer** |
| --- | --- | --- |
| **Participants approached directly (face to face, online via social media, or email and telephone)** | | |
| Students at Griffith University | ● | ● |
| Informal networks of friends and family residing in Australia | ● | ● |
| **Participants approached indirectly** | | |
| Griffith University volunteers' research broadcasts. | ● |  |
| **Social Media**  Griffith University internal mail; Griffith University Nutrition and Dietetics Students Canvas Site; PI and Co-PI’s LinkedIn, Research Gate, Facebook, and Twitter profiles. | ● |  |
| **Medical centres** (health centres, general practices, pharmacists, optometrists, physiotherapists, dentists, alternative therapy clinics, and veterinary clinics). |  | ● |
| **Community centres** (community health and social centre). | ● | ● |
| **Food outlets** (local grocery stores, cafes, restaurants, takeaways). |  | ● |
| **Private businesses (gyms, yoga centres, clothing stores, salons, shopping malls,** community boards). |  | ● |
| **Religious places** (church, temples, Gurudwara). |  | ● |
| **Leisure centres** (bowling clubs, sports clubs, rotary clubs). |  | ● |
| **Local radio stations (examples:** ABC Gold Coast, 94.1FM Gold Coast  Radio, Triple M Gold Coast 92.5) | | |

Table S4: Daily follow-up procedures in Phase 4 for monitoring tablet consumption and assessing perceived changes in overall food liking and appetite at the meal level.

| No. | Participants will be instructed to complete the following steps for each meal (breakfast, lunch, dinner, snack) consumed with the MB tablet: |
| --- | --- |
|  | Take a photograph of the meal before eating. |
|  | Dissolve the tablet in the mouth |
|  | Consume their meal |
|  | Take a photograph of the leftover meal. |
|  | Open the Qualtrics survey link (or the hard copy version of it) |
|  | Record the date and time of tablet intake |
|  | Report the main constituents of the meal (list of all main ingredients used). |
|  | Indicate whether that meal was breakfast, lunch, dinner or snack |
|  | Rate overall liking of the meal using the LAMS. |
|  | Indicate whether the tablet affected appetite (whether it helps them to eat more, or eat less, or has no effect) |
|  | Upload both pre-meal and post-meal photographs |

Table S5: Feasibility indicators, how and when they will be measured, and the possible reminders to be sent (if required).

| Feasibility indicator | Measurement methods | Time to measure/reminders(if) |
| --- | --- | --- |
| Recruitment success rate | - Number of participants recruited/eligible participants - Time taken to recruit the required number of study participants - Proportion of eligible study participants provided consent for the study (dropout rate before allocation)? | - Preintervention |
| Allocation success rate | - Group comparability in terms of number, age, sex, and PTC taste sensitivity - Factors challenged the attempt of equal allocation of study participants into two groups. | - At the start of the intervention (second visit) |
| Blinding adequacy | - Percentage of participants who do not know or guess whether they are in the MB or placebo group | - Postintervention (at 12 weeks) |
| Retention | - Percentage of participants completing the 12-week follow-up - Dropout rate - Reasons for dropouts (using open-ended questions) | - Post-intervention |
| Compliance | - Percentage of tablets (MB/placebo) taken   - Extracted from the Qualtrics survey report, where everyday study participants are expected to report every MB/placebo tablet taken). - Percentage of participants who rate their overall meal liking on HgLMS using the Qualtrics survey link - Percentage of participants who attach photos of meals (both before and after meal photos) | - Time: At each visit (at the 6^th^ week, 12^th^ week). - Reminder: However, compliance level will also be assessed weekly, and if at least one of the indicators (number of tablets taken, number of ratings provided, number of photos attached) drops below 75%*****, the principal investigator will send reminders to the participants. |
|  | - Percentage of participants who fill the 3-day food diary record sheet | - Time: At each visit (at the first week, 6th week, 12th week). - Reminder: A reminder will be sent one week before the next visit to remind participants to fill out the 3-day dietary record sheet. |
| Adverse effects | - Percentage of participants reported adverse effects related to intake of the tablets. - Percentage and frequency of each Grade of adverse effects reported   - Participants will be instructed to list any adverse effect that occurs throughout the intervention, record whether it is associated with intake of the tables and indicate the degree of severity of the adverse effect, i.e., Grade 0 (not described), Grade 1 (mild), Grade 2 (moderate), or Grade 3 (severe), as used by López-Plaza et al. (2024). (Supplementary material: Adverse effect recording sheet). | - Time: Throughout the intervention period, from the second home visit till the end of the intervention. - Reminder: Participants can report any Grade of adverse effect on any follow-up day and decide whether to continue or exit the study. However, if participants report Grade 3 adverse effects, the PI will ask them to exit the study, and the anonymous data collected until that point will be used for analysis. |
| *All compliance measures will be evaluated at each visit. With participants having low compliance (<75%), a detailed discussion on potential strategies to improve compliance levels (frequent text reminders and phone calls) for the subsequent visits will be done. This compliance threshold is based on a previous prospective interventional study on macronutrient prescription, in which the compliance level for high protein intake supplements was set at 75% (Campos-Nonato et al. 2017). | | |

**References**

Campos-Nonato, I., Hernandez, L., & Barquera, S. (2017). Effect of a high-protein diet versus standard-protein diet on weight loss and biomarkers of metabolic syndrome: a randomized clinical trial. *Obesity facts*, *10*(3), 238-251.
