# Supplementary material for: Effect of Miracle Berry on Taste Modification Properties Among Adults Living in Australia: A Multi‐Phase Study Protocol
Source: Food Sci Nutr. 2026 Mar 12;14(3):e71640. doi: 10.1002/fsn3.71640 (PMC13093657; doi:10.1002/fsn3.71640)
Supplement: Supplementary file 2 — File S2: Adverse‐effects monitoring and documentation procedures used during Phase 4 follow‐up. [file FSN3-14-e71640-s001.docx]

**Adverse effect recording sheet**

**Instructions**

As part of this study, we must monitor any potential adverse effects you may experience during the intervention period while taking the study tablets, regardless of whether the symptoms are related to the tablets or not. This will help us understand the effects of the intervention. You are encouraged to record any adverse symptoms during the intervention period. Common gastrointestinal symptoms associated with any product intake are listed in the table.

Please indicate the severity using the following scale (mark ‘**√**’ in the provided space)

- **Grade 0 (Not noticeable):** No symptoms observed.
- **Grade 1 (Mild):** Symptoms are noticeable but do not interfere with daily activities.
- **Grade 2 (Moderate):** Symptoms cause some interference with normal activities but are manageable.
- **Grade 3 (Severe):** Symptoms significantly interfere with your ability to perform daily activities.

For each symptom, please also indicate whether you believe it is related to the intake of the study tablet. In the column "Associated with taking the tablet," mark **‘1’** If you believe the symptom is directly related to the tablet intake, and mark **‘2’** If you do not believe the symptom is related to the tablet intake. Also, indicate whether that specific symptom is getting better (mark 1), remains the same (mark 2), or is getting worse (mark 3).

**Additional Information:** If you experience any adverse effects not listed in the table, document them in the provided space, including their severity, duration, and whether they are associated with tablet intake.

**Frequency of Reporting:** You will be expected to report symptoms regularly at each visit. Please document any changes in the severity or onset of symptoms and report any new symptoms as soon as they appear. However, if you face adverse effects related to tablet intake, with the severity of Grade 3 and above, please report immediately to the principal investigator.

Table: Adverse events recording sheet

| Symptoms | Grade of severity | | | | Associated with taking the table   1. Yes 2. No | Duration of the symptom (for how long it stayed, number of days) | Perception of symptoms   1. Getting better 2. The same 3. Getting worse |
| --- | --- | --- | --- | --- | --- | --- | --- |
|  | Grade 0 (Not detected) | Grade 1 (mild) | Grade 2 (moderate) | Grade 3 (severe) |  |  |  |
|  |  |  |  |  |  |  |  |
|  |  |  |  |  |  |  |  |
|  |  |  |  |  |  |  |  |
|  |  |  |  |  |  |  |  |
|  |  |  |  |  |  |  |  |
|  |  |  |  |  |  |  |  |
|  |  |  |  |  |  |  |  |
|  |  |  |  |  |  |  |  |
|  |  |  |  |  |  |  |  |
|  |  |  |  |  |  |  |  |
|  |  |  |  |  |  |  |  |
|  |  |  |  |  |  |  |  |
|  |  |  |  |  |  |  |  |
|  |  |  |  |  |  |  |  |
|  |  |  |  |  |  |  |  |
|  |  |  |  |  |  |  |  |
|  |  |  |  |  |  |  |  |
|  |  |  |  |  |  |  |  |
|  |  |  |  |  |  |  |  |
|  |  |  |  |  |  |  |  |
|  |  |  |  |  |  |  |  |
|  |  |  |  |  |  |  |  |
|  |  |  |  |  |  |  |  |
|  |  |  |  |  |  |  |  |
